# Enabling rapid and accurate grand discrimination of flue-cured tobacco: a near-infrared hyperspectral and machine learning approach

**DOI:** 10.3389/fpls.2026.1756218

**Published:** 2026-02-24

**Authors:** Jiang Zou, Hongbo Gao, Duo Wang, Yunquan Chen, Shiyou Deng, Nuo Shi, Shengjie Yang, Chunlin Huang, Dingchun Zi, Yu Du, Yuxiang Bai, Na Wang, Ge Wang, Zhengling Liu, Junhua Zhang, Peng Zhou

**Affiliations:** 1 Kunming University of Science and Technology, Kunming, Yunnan, China; 2 Yunnan Agricultural University, Kunming, Yunnan, China; 3 Kunming Branch of Yunnan Tobacco Company, Kunming, Yunnan, China

**Keywords:** characteristic bands, chemical analysis, machine learning, mantel test correlation analysis, near-infrared hyper spectroscopy, tobacco leaf grading

## Abstract

To address the inefficiency and subjectivity of manual grading, this study established a machine learning model based on near-infrared hyperspectral data (950–1650 nm) for the accurate classification of first-roasted tobacco grades. Multivariate statistical analysis uncovered the intrinsic correlations among grade, spectral data, and chemical composition, thereby laying a theoretical foundation for hyperspectral-based grading technology. Three preprocessing methods (namely, multiplicative scatter correction (MSC), standard normal variate transformation, and Savitzky–Golay convolutional smoothing) and four classification models (namely, random forest, backpropagation neural network, extreme learning machine, and partial least squares–discriminant analysis (PLS-DA)) were employed. Moreover, characteristic bands were selected through the successive projections algorithm (SPA) and competitive adaptive reweighted sampling to investigate how the number of characteristic bands affects the grade classification accuracy. The results showed that rank exhibited highly significant correlations with nicotine, reducing sugars, total sugars, and sugar-nicotine ratio, and that spectra exhibited highly significant correlations with nicotine. The classification accuracy of full-band MSC preprocessing combined with the PLS-DA model reached 98.5%, while the classification accuracy reached 94.0% when using 70% of the full bands selected using the SPA. In conclusion, near-infrared hyperspectroscopy combined with machine learning not only offers high efficiency, accuracy, and non-destructiveness in the grading of first-roasted tobacco leaves but also provides a theoretical basis for industrial hyperspectral grading by elucidating the correlations among spectrum, chemical composition, and grade. This method avoids the subjectivity of manual grading and offers key technical support to advance the intelligence and automation of first-roasted tobacco leaf grading in the tobacco industry.

## 1 Introduction

Tobacco is one of the most important cash crops worldwide, with an annual production exceeding 6.66 million tons. China alone produces more than 2 million tons annually, making it one of the world’s leading tobacco producers (Food and Agriculture Organization of the United Nations, 2025). In China, according to GB 2635–1992, first-roasted tobacco is classified into 42 grades based on maturity, leaf structure, oil content, color intensity, and other appearance traits. The grade of a first-roasted tobacco leaf determines its purchase price and industrial application, which directly influences the economic efficiency of the tobacco industry and the income of tobacco farmers (Marzan and Ruiz, 2019). There is no obvious difference in the appearance of similar grades of first-roasted tobacco, yet there is a substantial difference in their purchase price. For example, according to the 2024 purchase prices issued by the China Tobacco Monopoly Bureau, grade B1F was priced at 45.9 yuan/kg, while grade B2F was priced at 38.3 yuan/kg. Due to differences in the physical properties and intrinsic chemical compositions of various tobacco grades, it is necessary to classify raw materials by grade to meet the requirements of different tobacco products (Lu et al., 2021b). High-grade tobacco has higher mechanical strength, more balanced chemical composition, and better flavor, making it suitable for use as the primary raw material. In contrast, low-grade tobacco is mostly used as a filler. The misclassification of first-roasted tobacco grades may result in fluctuations or a decline in cigarette quality. Therefore, the accurate assessment of first-roasted tobacco leaf grades is of great importance.

Traditional tobacco grading mainly relies on sensory evaluation. Grading personnel require extensive training to master the relevant skills, and skilled evaluators are relatively scarce. Moreover, the accuracy of grading can be influenced by the experience of the personnel, environmental conditions, physical factors, and other variables. Online, real-time, and rapid tobacco grading during tobacco acquisition and processing is essential for adapting to large-scale production in the tobacco industry while reducing the labor intensity for workers (Lu et al., 2023a; Xin et al., 2023). Thus, the development of grading strategies for first-roasted tobacco leaves that do not rely on sensory evaluation has become a prominent research topic in the tobacco industry. The first method establishes decision rules based on fuzzy mathematics. For example, Zhang and Zhang, 2011 applied digital image processing with fuzzy set theory for the automatic classification of tobacco leaves. The second approach employs computer vision techniques supported by machine learning algorithms, including both traditional machine learning and deep learning methods (Lou and Zhang, 2018; Harjoko et al., 2019; Marzan and Ruiz, 2019; Li et al., 2021). Setiawan and Purnama, 2020 used the DarkNet19 algorithm to classify tobacco leaf images as healthy, curly, or hollow. However, both methods have certain limitations. Decision rule methods based on fuzzy mathematics require a class recognition model established using a large number of sample images. The mathematical derivation of this method is complex, and the image acquisition process is susceptible to interference, resulting in low classification accuracy and efficiency. However, computer vision techniques based on machine learning rely on high-quality, accurately labeled image data. The data acquisition equipment is susceptible to environmental conditions, leading to high annotation costs. Moreover, due to the minimal differences in appearance between tobacco leaves from adjacent producing areas and of similar grades, computer vision technology faces considerable challenges in achieving accurate classification, thereby imposing certain limitations on both methods (Niu et al., 2022).

Spectroscopic techniques are more accurate and environmentally adaptable than fuzzy mode and machine vision techniques. Furthermore, using spectroscopic techniques, we can obtain spectral curves that reflect the structural characteristics of the tobacco leaf as well as chemical indices and internal structural information closely related to leaf quality (Zhang et al., 2023), providing more comprehensive information about the samples. Due to the advantages of machine learning in generalization capability, computational speed, and the handling of high-dimensional data, the combination of spectroscopy with machine learning has yielded encouraging results in tobacco classification (Moreira et al., 2009; Li et al., 2020; Qin et al., 2023). However, traditional spectroscopic methods (e.g., multispectral technology and near-infrared spectroscopy) have a limited number of bands, and interference with a key band can compromise the entire analysis. When the sample composition or morphology is highly similar, traditional spectroscopy struggles to differentiate samples due to the smaller number of bands and low-dimensional features. Hyperspectral methods avoid these drawbacks due to their extremely high spectral resolution and continuous spectral band coverage, making them a superior alternative in various applications (Hu et al., 2021; Wu et al., 2025).

Hyperspectral techniques, which integrate image and spectral information, have been applied in tobacco research (including maturity detection, chemical composition analysis, and pest and disease monitoring) (Lu et al., 2023b; Yang et al., 2025), and near-infrared hyperspectral technology, in particular, finds extensive application throughout the entire tobacco industry chain for quality control—enabling rapid screening of basic leaf indices during procurement to replace manual labor and improve efficiency, facilitating real-time monitoring of key chemical components in cut tobacco and lamina during processing to ensure consistency, and supporting field-based monitoring of tobacco growth to inform breeding and cultivation optimization (Marcelo et al., 2019; Chen et al., 2021; Zhang et al., 2023). However, this technology also faces several well-recognized complexities and challenges, including but not limited to the substantial storage demands due to high-dimensional data, high costs associated with data processing and computation, as well as issues related to model complexity and interpretability (Luo et al., 2024; Wang et al., 2024; Panda et al., 2025). Dante and Sahu, 2018 applied linear discriminant analysis to visible–near-infrared hyperspectroscopy (400–1000 nm) to model roasted and white-ribbed tobacco grades, representing an initial application of hyperspectral techniques in tobacco grade classification. Wei et al., 2024 employed a one-dimensional convolutional neural network (1D-CNN) model in combination with the least angle regression (LAR) algorithm to classify tobacco leaves into 10 grades. The essence of tobacco leaf grades is the external expression of the suitability and balance of their chemical compositions. Because the visible (400–750 nm) and near-infrared (750–1000 nm) two spectral ranges within the visible–near-infrared band exhibit weak correlation, spectral analysis may fail to fully capture the complex chemical features of tobacco leaves. This imposes certain limitations on the application of the visible–near-infrared band in tobacco leaf grade classification. The near-infrared band is more stable than the visible–near-infrared band and provides more comprehensive information on the chemical composition of tobacco. Therefore, the use of near-infrared hyperspectroscopy to classify tobacco grades and explore the relationship among tobacco grades hyperspectral data, and chemical composition is highly valuable for classifying tobacco grades.

Compared to basic preprocessing methods (e.g., standardization or centering) that mainly adjust data scale, the preprocessing methods adopted in this study include Multiplicative Scatter Correction (MSC), Standard Normal Variate (SNV) transformation, and Savitzky–Golay (SG) convolutional smoothing. These techniques effectively mitigate spectral interference, preserve intrinsic nonlinear relationships in hyperspectral data, and generate preprocessed spectra with improved compatibility for subsequent classification modeling (Roger et al., 2022; Yang et al., 2024). Unlike deep learning models such as CNNs or Transformers, which usually demand large annotated datasets and significant computational resources, classifiers including Random Forest (RF), Backpropagation Neural Network (BPNN), Extreme Learning Machine (ELM), and Partial Least Squares-Discriminant Analysis (PLS-DA) are more practical for limited-sample scenarios and cost-sensitive industrial applications (Zhang et al., 2022; Lu et al., 2023b). Thus, this study employs these four models (covering both linear and nonlinear approaches) to comparatively evaluate their performance and applicability in first-roasted tobacco leaf grading. Compared to Principal Component Analysis (PCA), Genetic Algorithm (GA), and stepwise regression, Competitive Adaptive Reweighted Sampling (CARS) and Successive Projections Algorithm (SPA) offer feature selection strategies better suited to first-roasted tobacco leaves’ spectral characteristics—key information resides in narrow reflectance bands. Thus, CARS and SPA are adopted to reduce data redundancy and enhance classification model robustness (Zhu et al., 2017; Huang et al., 2021).

Herein, multivariate statistical analysis was used to explore correlations among tobacco grades, spectral data, and chemical compositions. Then, using near-infrared hyperspectral data of first-roasted tobacco leaves, three preprocessing methods—MSC, SG, SNV—and four classification models (RF, ELM, BPNN, PLS-DA) were applied for tobacco grade classification to identify the optimal industrial model. Finally, characteristic wavelength selection algorithms examined model performance across different bands, balancing simplicity and predictive accuracy. Overall, this study provides a feasible strategy for lightweight deployment and industrial application of hyperspectral technology in tobacco leaf grading and is expected to enhance the economic benefits of the tobacco industry and lay a solid theoretical foundation for its practical industrial application.

## 2 Materials and methods

### 2.1 Reagents and materials

Nicotine standards (CAS:54-11-5, purity ≥ 98%) were purchased from Aladdin Biochemical Technology Co., Ltd. (Shanghai, China). D-glucose (CAS:50-99-7, purity ≥ 99.5%), sodium chloride (NaCl, CAS:7647-14-5, purity ≥ 99.5%), and acetic acid (HAc, analytical grade) were obtained from Sinopharm Chemical Reagent Co., Ltd. (Shanghai, China). The potassium single-element standard solution (GBW(E)080125) was purchased from the National Institute of Metrology, China (NIM).

### 2.2 Tobacco sample preparation

First-roasted tobacco leaves are primarily processed products obtained by curing harvested mature fresh leaves with artificial heat. In this study, such samples were collected from eight counties of Kunming City, Yunnan Province, during 2023 and 2024 [All samples were provided under the Research on the Science and Technology Plan Project of Kunming Branch of Yunnan Provincial Tobacco Company (KMYC202303)]. All samples were flue-cured tobacco of the species *Nicotiana tabacum* L. (genus *Nicotiana* L., family Solanaceae). In accordance with the Chinese National Standard GB 2635–1992, the tobacco leaf grades were classified based on leaf position, grade tier, and color (Standardization Administration of China, 1992). The lugs, cutters, and leaf terms correspond to the lower (X), middle (C), and upper (B) positions of leaves on the stalk, respectively. Each part is further divided into three or four grades (denoted as 1, 2, 3, and 4). The color categories include eight orange (F) and one lemon (L). Groups are then formed by combining the leaf position, grade, and color. For example, C1F indicates orange cutters grade one. All samples were graded by professional graders from China Tobacco Corporation, Kunming City, based on the above criteria. The dataset included nine major acquisition grades, comprising a total of 1,784 samples (see Appendix 1 for details).

### 2.3 Chemical constituent measurement

The K_2_O content was determined according to the Chinese Tobacco Industry Standard YC/T 173-2003: Tobacco and Tobacco Products–Determination of Potassium –Flame Photometry (State Tobacco Monopoly Administration, 2003). The chloride ion (Cl^−^) content was determined according to YC/T 162-2011: Tobacco and Tobacco Products–Determination of Chloride–Continuous Flow Method (State Tobacco Monopoly Administration, 2011). The total sugar (TS) and reducing sugar (RS) contents were determined according to YC/T 159-2019: Tobacco and Tobacco Products–Determination of Water-Soluble Sugars–Continuous Flow Method (State Tobacco Monopoly Administration, 2019). The nicotine (Nic) content was determined according to YC/T 468-2021: Tobacco and Tobacco Products–Determination of Total Alkaloids–Continuous Flow (Potassium Thiocyanate) Method (State Tobacco Monopoly Administration, 2021). After determining the contents of Nic, TS, K, and Cl, the sugar-nicotine ratio (S/N = TS/Nic) and the potassium–chloride ratio (K/Cl = K_2_O/Cl^−^) were calculated.

### 2.4 Multivariate statistical analysis

Principal component analysis (PCA) is commonly used to reduce the dimensionality of data, and the Mantel test is used to assess the correlation between matrices, with its significance evaluated via random permutations (Nekola and White, 1999; Hu et al., 2024). PCA transforms the original high-dimensional variables into a set of linearly uncorrelated low-dimensional variables, called principal components (PCs), using orthogonal transformation to extract the main source of variance. The data dimensionality is reduced and used as a basis for clustering algorithms (Patra et al., 2022). The Mantel test is a nonparametric statistical method that determines significance by calculating the correlation coefficients between two matrices, randomly permuting the ranks of one matrix to generate a random distribution, and comparing the observed correlation with this distribution. The Mantel test is used to assess the correlation between two distance matrices, offering the capability to handle inter-matrix relationships that cannot be evaluated using traditional correlation coefficients (Crabot et al., 2019). In this study, dimensionality reduction and clustering analyses of chemical constituents and hyperspectral data were performed using PCA, and the correlations among various chemical components, as well as the relationships between grade, spectral data, and chemical composition, were analyzed using the Mantel test.

### 2.5 Hyperspectral acquisition

#### 2.5.1 Hyperspectral imaging

**Figure 1:**
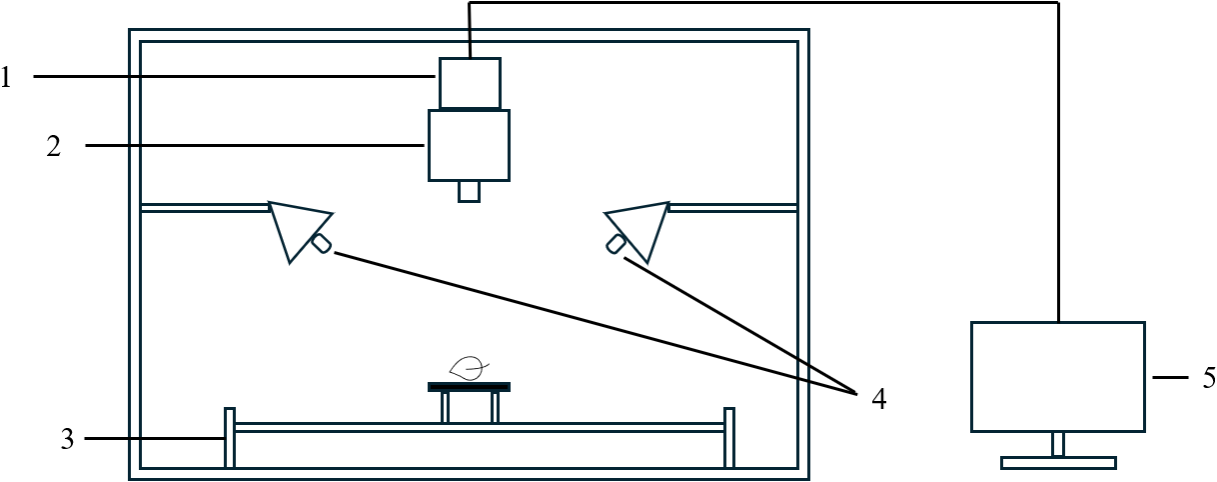
Schematic of the structure of the hyperspectral imaging system.

The hyperspectral data used in the experiment were collected using the GTM-900Pro tobacco comprehensive test bench (Chuangheyi Electronic Technology Development Co., Ltd. Shanghai, China). The GTM-900Pro consists of three modules: a sample visual inspection module, a near-infrared spectrometer (950–1650 nm, 351 bands), and the integrated industrial electronic control module (**Figure 1**).

1. Camera 2. Near infrared spectrograph 3. Scanning platform 4. Light source 5. Computer.

#### 2.5.2 Acquisition of hyperspectral images

During data acquisition, the ambient lighting was kept constant to avoid interference from external light sources. To minimize the adverse effects of temperature and humidity fluctuations on spectral data acquisition, the room temperature was strictly maintained at 25 ± 1 °C throughout the entire data acquisition process. Tobacco samples were placed horizontally on the inspection stage, and black and white reference plate corrections were applied to the raw hyperspectral images to minimize noise caused by factors such as illumination and camera current variations. A standard reflective whiteboard was placed vertically in front of the imaging lens, and a single frame of whiteboard data corresponding to the current slit was captured for calibration. By attaching a lens cover to the lens, the black and white reference correction can be performed by acquiring the corresponding dark frame data. The calculations are detailed in Equation 1:

(1) $$\begin{array}{c} R=\frac{I_{raw}-I_{dark}}{I_{white}-I_{dark}} \end{array}$$

where R indicates the corrected sample image, $I_{raw}$ denotes the original image of the sample, $I_{dark}$ signifies a blackboard correction image, and $I_{white}$ represents a whiteboard correction image.

#### 2.5.3 Spectral data extraction

During spectral data acquisition, the instrument measures the transmittance at 81 points of each sample, through 81 holes arranged in a 9×9 matrix and calculates the average of these measurements to represent the spectral data of the tobacco piece. In traditional machine learning, a 70:30 or 80:20 split between training and test sets is commonly used. A 70:30 division has the advantage of not requiring an independent validation set, providing more stable evaluation results that can reduce the risk of overfitting. Therefore, in this experiment, 70% of the samples were randomly selected from each level as the training set, and 30% as the test set (Toleva, 2021). The sample set in this study comprised flue-cured tobacco leaves collected from eight counties/districts in Kunming over two consecutive years (2023 and 2024), thus capturing geographical and interannual variability to a certain extent. In addition, an independent test set with 535 samples was established, which was completely independent of the training data and had a consistent grade distribution with the latter. Therefore, no additional external validation is required to confirm the credibility of the developed model (Godin et al., 2015).

### 2.6 Spectral data processing

Various environmental factors and individual differences among the test samples can interfere with the spectral data during collection. These interferences may reduce the accuracy of the collected data and introduce irrelevant information or noise into the samples, such as external light, electrical interference, and artificial transmission noise. These factors can affect model development and reduce the final predictive accuracy; therefore, the spectral data must be preprocessed before constructing the model. Commonly used spectral preprocessing methods include SG smoothing (for random noise removal), SNV transformation (to correct scattering-induced spectral distortions), and MSC (which eliminates spectral differences due to scattering intensity variations and enhances spectrum–reference data correlations) (Hu et al., 2025; Hao et al., 2025; Luo et al., 2026). Additionally, mean centering removes absolute absorption by subtracting the average spectrum, while orthogonal signal correction filters out spectral information orthogonal to the concentration matrix prior to multivariate calibration (Qu et al., 2005; Yan, 2025). This process simplifies the model and improves its predictive ability (Rinnan et al., 2009). In this study, the spectral data were preprocessed using three methods: MSC, SNV, and SG.

### 2.7 Feature selection algorithms

The large sample size and numerous spectral bands in hyperspectral data often lead to data redundancy and high dimensionality, which can result in a decline in both the accuracy and efficiency of spectral model classification. To address this issue, important feature wavelengths can be extracted using a feature band selection algorithm to reduce data dimensionality, improve model accuracy, and reduce the risk of overfitting (Medjahed and Ouali, 2018). The SPA and CARS algorithms were chosen as feature selection methods for the experiments. The SPA is a forward variable selection method used to extract characteristic wavelengths from spectral data while minimizing multicollinearity among variables (Soares et al., 2013). The algorithm iteratively selects new bands with the lowest linear correlation to the previously chosen band. In each iteration, the remaining bands are projected onto the orthogonal complementary space of the selected band. The band with the maximum projection vector length is selected, and the final set of characteristic wavelengths is determined according to the model performance. CARS is a feature variable selection algorithm inspired by Charles Darwin’s “survival of the fittest” theory (Wang et al., 2018). The algorithm combines Monte Carlo sampling with PLS regression coefficients to identify feature bands that contribute significantly to the prediction model by simulating a process of competition, elimination, and iterative optimization (Li et al., 2009).

### 2.8 Classification model

Near-infrared hyperspectral data have high dimensionality and subtle differences in the spectral features among sample categories. Traditional statistical methods struggle to effectively differentiate overlapping spectral features, while classification models (e.g., CNNs, support vector machines, and orthogonal PLS-DA) can enhance classification performance by automatically extracting deeper features from high-dimensional data (Sun et al., 2021). In this study, four algorithms (namely, RF, BPNN, ELM, and PLS-DA) were used to build the classification model. RF is a meta-estimator based on ensemble learning, which implements classification decisions by aggregating the predictions of multiple classification and regression trees through a voting mechanism (Breiman, 2001). During the construction of decision trees, the observations (rows) and variables (columns) are chosen randomly, and the trees are grown without pruning. In categorization, each sample is passed through all decision trees, and the predicted category labels from each tree are counted. The category with the most votes is selected as the categorization result (Cutler et al., 2007). The BPNN model is a supervised learning model based on the error backpropagation algorithm, which can capture complex patterns in data through multilayer nonlinear transformations. The network typically consists of an input layer, a hidden layer, and an output layer. Learning is achieved by feeding the neural network output back to the hidden layers and adjusting the weights and thresholds to minimize the total error (Fu and Tian, 2020). The ELM model is an efficient single hidden layer feedforward neural network consisting of input, hidden, and output layers. The algorithm reduces training complexity by randomly initializing the hidden layer parameters and analytically solving for the output weights. Specifically, the optimal solution is obtained by randomly generating the input weights (w) and hidden layer biases (b), setting the activation function g(x) and the number of neurons in the hidden layer (L), and calculating the output weights (β) between the hidden and output layers (Yang et al., 2018). PLS-DA combines PLS and discriminant analysis, providing an effective approach for high-dimensional and multicollinear datasets. The core idea of PLS-DA is to maximize the covariance between independent variables and class labels by projecting the original variables onto a low-dimensional latent space, extracting the most discriminative features for classification. Compared with traditional classification models, such as logistic regression and support vector machines, PLS-DA effectively mitigates the “curse of dimensionality” through dimensionality reduction while retaining key discriminative information in the data. High-dimensional data are represented in a lower-dimensional space using a set of latent variables. The latent variables provide an optimal representation of the predictive data X, maximizing their predictive power for the response data Y (Barker and Rayens, 2003). PLS-DA can provide a model by identifying the correlation between the scores of X and Y (Chandra and Kundu, 2024). The X and Y data are processed separately in the PLS-DA external model, while internal relationships establish connections between the two datasets. The external relationships among the predictor variables, response variables, and their corresponding scores and loading matrices in the latent dimensions are formally defined in Equations 2–5 as follows:

(2) $$\begin{array}{c} X=t_{1}p_{1}^{T}+t_{2}p_{2}^{T}+\cdots +t_{n}p_{n}^{T}+E=TP^{T}+E \end{array}$$

(3) $$\begin{array}{c} Y=u_{1}q_{1}^{T}+u_{2}q_{2}^{T}+\cdots +u_{n}q_{n}^{T}+F=UQ^{T}+F \end{array}$$

where t and u represent the principal component (PC) score matrices of X and Y, respectively; p and q are load vectors that indicate the dominant directions by maximizing the covariance within the X and Y data. The errors E and Fcancel out to zero if each dimension of X and Y is considered. The relationship between the PC scores T and U represents the internal model linking X and Y.

(4) $$\begin{array}{c} U=TB \end{array}$$

where B is the regression matrix. Y can be written as:

(5) $$\begin{array}{c} Y=TBQ^{T}+F \end{array}$$

The relationship equations are derived iteratively, using newly calculated residuals to update the scores and loadings, until the residuals become negligible or the number of PLS-DA latent variables exceeds the number of X variables. The percentage of variance explained and cross-validation residuals are used to determine the number of PLS-DA latent variables.

### 2.9 Evaluation of model performance

#### 2.9.1 Accuracy

The test set accuracy reflects the ability of a model to generalize to unseen data and serves as a key performance indicator (Liu and He, 2025). The test set accuracy is the probability of correctly predicting an outcome across all test set samples, which is calculated as shown in Equation 6:

(6) $$\begin{array}{c} Acc=\frac{TP+TN}{TP+TN+FP+FN}\times 100\% \end{array}$$

where TP denotes the number of correctly categorized positive samples; TN denotes the number of correctly categorized negative samples; FP denotes the number of negative samples incorrectly categorized as positive samples; and FN denotes the number of positive samples incorrectly categorized as negative samples.

#### 2.9.2 Confusion matrix

A confusion matrix, also known as a likelihood or error matrix, is an important tool for comparing the classification results with actual values. It effectively represents the accuracy of the classification results and is widely used to evaluate classifier performance (Theissler et al., 2022).

### 2.10 Software

The multivariate statistical analyses used in this study, including PCA and the Mantel test (Pearson), were performed using the Metware Cloud platform (https://cloud.metware.cn/). The MSC, SNV transformation, and SG; RF, BPNN, ELM, PLS-DA classification models; and SPA and CARS feature band algorithms were implemented using MATLAB 2023b. All experiments were conducted on a computer running Windows 11, equipped with an AMD Ryzen 9 7845HX CPU, 16 GB DDR5 RAM (2×8 GB), and an NVIDIA RTX 4070 Laptop GPU.

## 3 Results and discussion

### 3.1 Morphological characteristics of tobacco leaf samples

**Figure 2:**
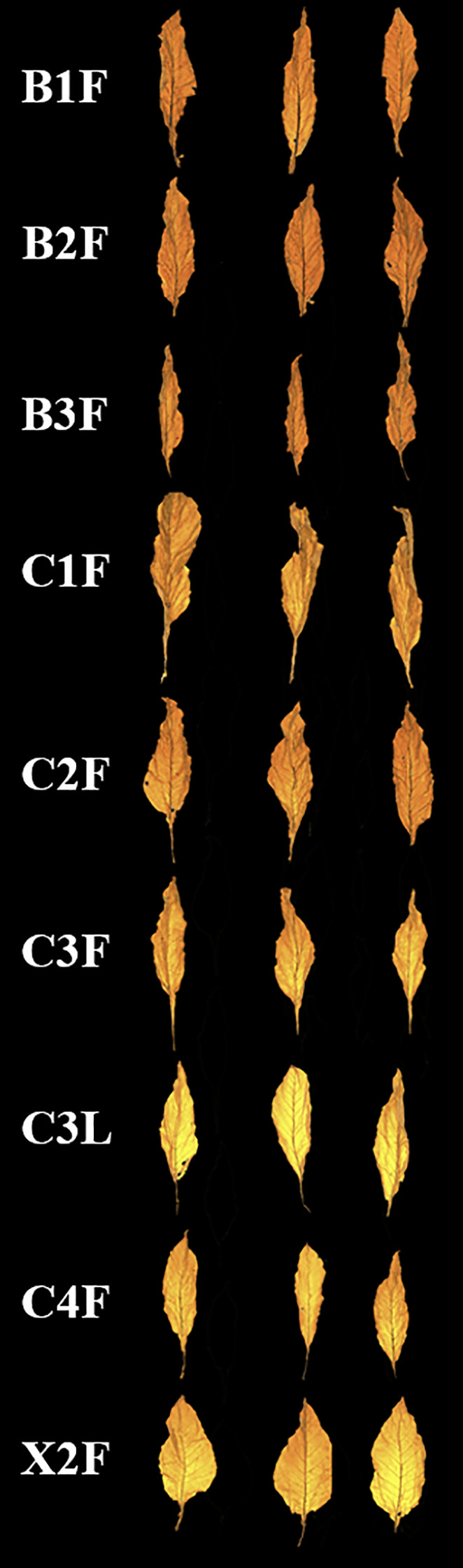
Photograph showing various tobacco samples.

A total of nine commonly used tobacco leaf grades (B1F, B2F, B3F, C1F, C2F, C3F, C3L, C4F, and X2F) were collected, and their images were acquired using a near-infrared hyperspectral imaging system for subsequent analysis (**Figure 2**). The upper leaves exhibit thicker and more prominent veins, a broader shape with a sharper tip, and medium to high thickness. The middle leaves have moderately developed veins, a slightly curved tip, a broader shape with a blunter tip, and medium to slightly thin thickness. The lower leaves display finer veins, a broader, more rounded shape, and slightly thin to thin thickness. Grade 1 tobacco is characterized by high maturity, a loose leaf structure, high oil content, and deep coloration. Grade 2 tobacco has good maturity, a firm leaf structure, moderate oil content, and strong coloration. Grade 3 tobacco exhibits average maturity, a slightly dense leaf structure, low oil content, and moderate coloration. The morphological characteristics of the tobacco grades can be described as follows: B1F: high maturity, firm leaf structure, slightly high thickness, high oil content, and deep coloration; B2F: high maturity, firm leaf structure, slightly high thickness, moderate oil content, and strong coloration; B3F: high maturity, slightly dense leaf structure, slightly high thickness, moderate oil content, and moderate coloration; C1F: high maturity, loose leaf structure, medium thickness, high oil content, and deep coloration; C2F: high maturity, loose leaf structure, medium thickness, moderate oil content, and strong coloration; C3F: high maturity, loose leaf structure, medium thickness, moderate oil content, and moderate coloration; C3L: high maturity, loose leaf structure, slightly thin thickness, moderate oil content, and moderate coloration; C4F: high maturity, loose leaf structure, slightly thin thickness, low oil content, and moderate coloration; and X2F: high maturity, loose leaf structure, slightly thin thickness, low oily content, and moderate coloration. There are significant differences in the vein structure, leaf shape, and thickness of different parts of the tobacco leaves, which can be used to differentiate the various leaf positions (**Figure 2**). However, when comparing the same part of different grades of tobacco, such as B1F vs. B2F or C1F vs. C2F, the leaves exhibit similar maturity, structure, and thickness, only differing in oil content and coloration. Leaf structure and thickness differ only when there is a large difference in grades, such as B1F vs. B3F or C1F vs. C4F.

### 3.2 Tobacco chemical composition analysis

#### 3.2.1 Chemical composition of different grades of tobacco leaves

The contents of nicotine (Nic), Cl^−^, K_2_O, reducing sugar (RS), and total sugar (TS) in different tobacco grades were determined, and the corresponding sugar-nicotine ratio (S/N) and potassium-chloride ratio (K/Cl) were calculated (**Table 1**). As the primary contributor to the strength and satisfaction of tobacco products, the Nic content directly determines the core quality attributes of the product. Consequently, variations in Nic content across leaf positions and grades are essential considerations in the formulation of tobacco products (Darkis and Hackne, 1952). The Nic content of the upper leaves was significantly higher than that of the middle and lower leaves; however, the differences in the Nic content of B1F, B2F, and B3F were minimal. These differences can be precisely used in tobacco product formulations to meet different quality requirements. For high-end flue-cured cigarettes with a rich and strong impact, the formula prioritizes the use of upper leaves, leveraging their high Nic content to enhance smoking satisfaction and form the core flavor profile. B3F leaves are specifically incorporated into products such as low-tar cigarettes, providing a lower tar content while maintaining smoking satisfaction through their high nicotine content (McKinney et al., 2014). RS and TS are positively correlated with the smoothness of smoke; in particular, fructose and glucose can balance irritation by regulating the production of acidic substances. This property makes the differences in sugar content among leaf components and grades the core means for regulating smoke palatability in tobacco product formulation (Stepanov et al., 2025). The RS and TS contents of the lower leaves were significantly higher than those of the middle and upper leaves, with C3L and X2F exhibiting significantly higher RS and TS levels than the other grades. These differences are precisely applied in tobacco product formulations to achieve specific smoke quality objectives. For mid-to-low-end flue-cured cigarettes characterized by mildness and low irritation, the formula incorporates a larger proportion of lower leaves with high sugar content. It leverages the high RS and TS contents of lower leaves to enhance smoke smoothness, reinforcing the effect of balancing acidic substances and reducing irritation. For mid-range products aiming for moderate sweetness and smoothness without blandness, middle leaves can serve as the main component, blended with an appropriate amount of X2F-grade tobacco leaves. This not only ensures the sweet flavor of the smoke but also balances the taste layers and reduces irritation (Talhout et al., 2006). The S/N ratio directly affects the sensory acceptability and smoking behavior of tobacco by modulating the synergistic effects of Nic stimulation and sugar sweetness. This makes the variations in S/N ratios across leaf positions and grades a major consideration in the design of tobacco products (Roemer et al., 2012). The lower leaves had significantly higher S/N ratios than the middle or upper leaves, with C3L and X2F exhibiting significantly higher S/N ratios than the other grades. These differences are systematically used in the formulation of tobacco products. For flue-cured cigarettes with a mild, sweet, and low-irritation taste, the formula prioritizes tobacco leaves with a high S/N ratio, leveraging their strong synergistic effect to neutralize the irritation of smoke and enhance sensory acceptability (Nikolova et al., 2021). K_2_O and Cl^−^ are key regulators of tobacco combustibility: K^+^ enhances combustibility by lowering the ignition temperature and promoting cellulose decomposition, while Cl^−^ inhibits combustion speed. Thus, the K/Cl ratio serves as a critical determinant of tobacco combustibility, with ratios exceeding 5 enhancing combustion and values below 1 potentially inhibiting ignition. The variations in K_2_O, Cl^−^, and K/Cl ratio among leaf components and grades serve as the core basis for the precise regulation of tobacco combustibility in product formulation (Myhre et al., 1956). X2F had the highest K_2_O content, while C1F had the highest Cl^−^ content. The K/Cl ratios of B1F, C2F, C3F, C3L, and C4F were comparable and significantly higher than those of the other grades. However, there was no significant difference in K/Cl ratios among the three leaf positions. These differences are strategically applied in tobacco product formulations to meet various combustion requirements. For high-end flue-cured cigarettes with easy ignition, even combustion, and white ash, the formula prioritizes the use of X2F, B1F, and C3L tobacco leaves while strictly controlling the inclusion of C1F-grade tobacco leaves. This ensures that the overall K/Cl ratio of the leaf blend remains above 5, thereby providing a smoking experience with smooth smoke release and minimal risk of extinguishment (Zhong, 2019). These differences in chemical composition not only distinguish the grades of first-roasted tobacco but also form the core basis for the design of tobacco product formulations in terms of product style, core quality, and consumer preferences.

**Table 1:** Contents of nicotine (Nic), reducing sugar (RS), total sugar (TS), K_2_O, and Cl^−^, as well as the sugar-nicotine ratio (S/N) and potassium-chloride ratio (K/Cl), for different tobacco leaf grades.

Grade	Nic (%)	RS (%)	TS (%)	K_2_O (%)	Cl^−^ (%)	S/N	K/Cl
B1F	2.66 ± 0.71b	23.30 ± 4.65f	25.50 ± 5.09d	2.48 ± 0.58b	0.14 ± 0.07e	10.70 ± 4.64e	21.10 ± 9.87a
B2F	2.77 ± 0.68a	23.10 ± 4.66f	24.70 ± 4.61d	2.27 ± 0.47de	0.17 ± 0.10de	9.75 ± 4.00e	18.10 ± 9.39b
B3F	2.67 ± 0.53ab	22.90 ± 5.12f	23.30 ± 4.98e	2.13 ± 0.57f	0.19 ± 0.10cd	9.23 ± 3.21e	14.80 ± 9.44c
C1F	2.07 ± 0.55c	25.40 ± 2.84d	27.50 ± 3.23bc	2.23 ± 0.54ef	0.30 ± 0.18a	14.40 ± 4.95d	11.90 ± 9.97d
C2F	1.91 ± 0.58d	26.60 ± 4.60c	28.50 ± 5.09b	2.45 ± 0.65b	0.23 ± 0.19b	16.80 ± 7.45c	19.80 ± 14.00ab
C3F	1.54 ± 0.43f	25.00 ± 5.53e	26.90 ± 6.56c	2.29 ± 0.59cde	0.19 ± 0.15cd	19.10 ± 7.90b	20.40 ± 16.70ab
C3L	1.36 ± 0.47g	30.10 ± 7.23a	33.00 ± 7.46a	2.40 ± 0.65bc	0.20 ± 0.16bc	28.70 ± 17.81a	21.50 ± 21.10a
C4F	1.67 ± 0.50e	24.80 ± 5.91e	28.00 ± 6.83bc	2.36 ± 0.57bcd	0.16 ± 0.10de	19.10 ± 9.13b	18.80 ± 9.83ab
X2F	1.25 ± 0.34h	29.00 ± 4.62b	32.60 ± 5.00a	2.81 ± 0.62a	0.21 ± 0.13bc	28.70 ± 10.96a	18.00 ± 9.39b
Part							
Upper leaves	2.70 ± 0.65a	23.10 ± 4.81c	24.50 ± 4.98c	2.29 ± 0.56bc	0.17 ± 0.09b	9.90 ± 4.04c	18.00 ± 9.90a
Middle leaves	1.71 ± 0.57b	28.00 ± 6.17b	28.80 ± 6.40b	2.35 ± 0.61bc	0.22 ± 0.17a	21.60 ± 11.50b	18.50 ± 15.30a
Lower leaves	1.25 ± 0.34c	29.00 ± 4.62a	32.60 ± 5.00a	2.81 ± 0.62a	0.21 ± 0.13a	26.70 ± 10.96a	18.00 ± 9.39a

Lowercase letters indicate significant differences in the chemical composition of the raw tobacco leaves (P < 0.05).

#### 3.2.2 Principal component analysis of chemical composition of tobacco leaves

**Figure 3:**
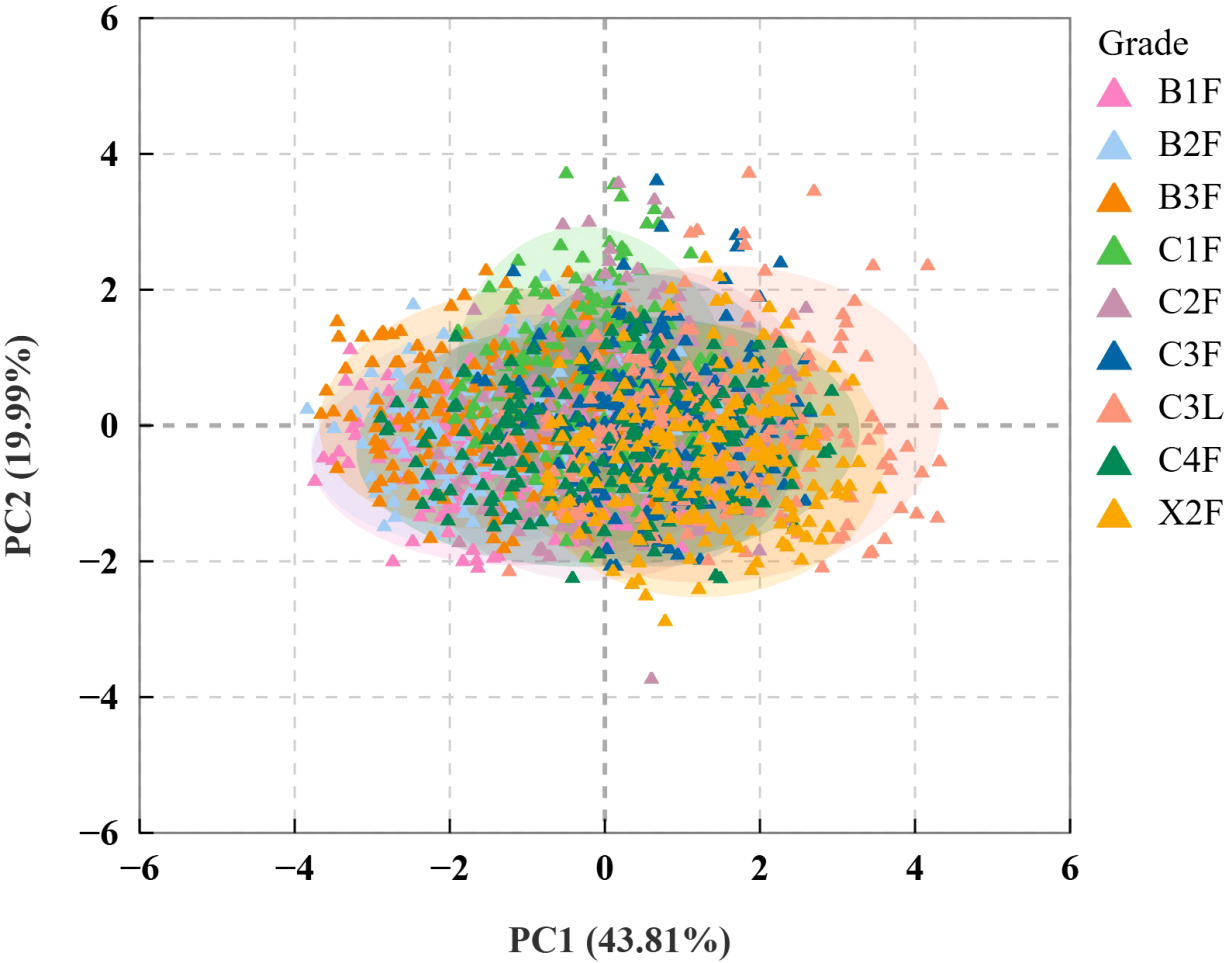
PCA score plot of chemical composition across first-roasted tobacco leaf grades.

PCA was performed on the chemical components of the collected tobacco leaves (**Figure 3**), revealing that the first two principal components cumulatively accounted for 63.8% of the total variance (PC1, 43.81%; PC2, 19.99%). Despite PC1 and PC2 explaining 63.8% of the total variance, leaves of different grades could not be clearly distinguished, likely due to the low dimensionality of the chemical composition data and the fact that tobacco grades are primarily differentiated based on physical characteristics such as maturity, leaf structure, thickness, and oil content. Although chemical composition underlies the physical appearance of tobacco and can influence it to some extent, the relationship between the two is complex. Therefore, chemical composition alone cannot be directly used to differentiate tobacco grades.

### 3.3 Spectral analysis of tobacco leaves

#### 3.3.1 Tobacco leaf spectrum

**Figure 4:**
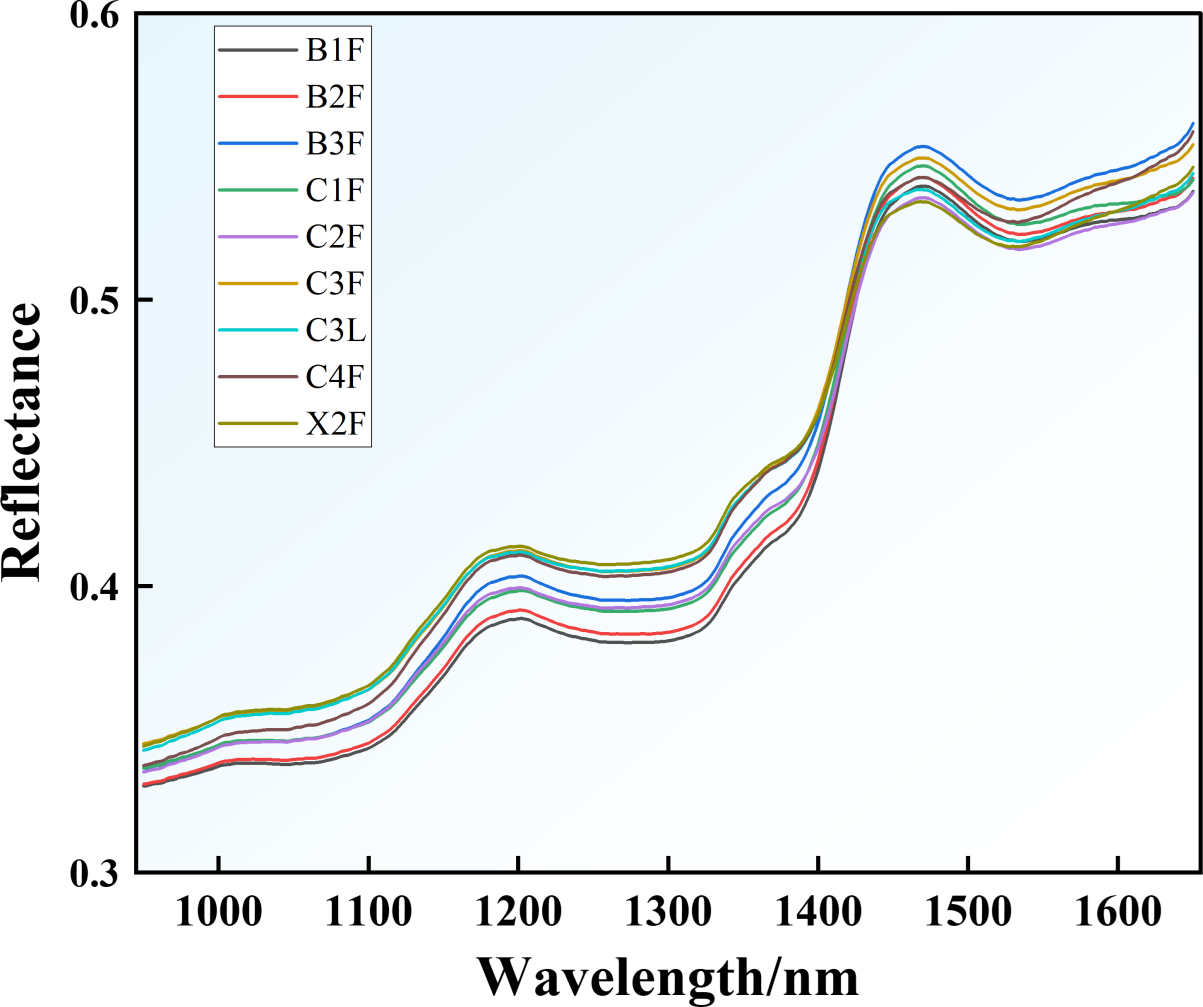
Average reflectance spectra of different tobacco leaf grades.

The spectra of nine tobacco grades were scanned in the range of 950–1650 nm, and their average values were calculated, with each spectrum consisting of 351 bands (**Figure 4**). The reflectance spectra of the tobacco leaves are generated through the interaction between light and the leaf tissue. The spectra exhibit distinct peaks at approximately 1180 and 1470 nm. The second overtone or combination frequency vibrations of C–H bonds in fructose, glucose, and portions of cellulose and pectin in flue-cured tobacco leaves give rise to a characteristic reflectance peak at 1180 nm (Wu et al., 2021; Liu et al., 2024). Additionally, surface shrinkage and texture homogenization in flue-cured tobacco leaves enhance the scattering effect at 1180 nm, further contributing to the characteristic reflectance peak. In addition, the low moisture content of flue-cured tobacco leaves reduces O–H bond absorption in water, resulting in a relative increase in reflectance near 1470 nm (Zhang and He, 2011; Liu et al., 2023).

A comparison of the average reflectance spectra (**Figure 4**) of nine grades of first-roasted tobacco revealed that the spectra of B1F and B2F were similar; those of B3F, C1F, and C2F were similar; and those of C3F, C3L, C4F, and X2F were similar. Aside from slight differences in color intensity and oil content, the appearance characteristics of B1F and B2F first-roasted tobacco leaves are largely consistent, resulting in minimal spectral variation and similar spectral profiles. The spectral curves of tobacco leaves of different grades show that the spectral reflectance of B1F, B2F, C1F, and C2F is lower than that of B3F, C3F, and C4F within the range of 1000–1400 nm. This is because B1F, B2F, C1F, and C2F have higher maturity and a looser leaf structure. In addition, B3F contains lower levels of RS and TS than B1F and B2F, while C1F and C2F have significantly higher nicotine content than C3F and C4F. These differences result in variations in the spectral reflectance of hydrogen-containing functional groups such as C–H and O–H, ultimately causing differences in the reflectance spectra between B1F, B2F, C1F, C2F and B3F, C3F, C4F within the range of 1000–1400 nm (Lu et al., 2023b).

From a molecular spectroscopy perspective, these observed spectral differences stem from the overtone and combination vibrations of hydrogen-containing functional groups, directly tied to leaf chemistry (Yang et al., 2015). Within this band, the spectral features of key chemical components in tobacco leaves stem from the vibrations of specific chemical bonds. For carbohydrates (e.g., RS and TS), characteristic reflectance originates from O–H stretching-bending combination bands and C–H vibrations. Higher sugar content generally results in weak reflectance at 1200–1300 nm and 1400–1500 nm, lowering overall spectral reflectance levels (Soares et al., 2019). For Nic, its molecular functional groups (pyridine ring, N–CH_3_) exhibit significant absorption at 1100–1200 nm and 1300–1400 nm due to C–H and N–H vibrations (Yang et al., 2015). Consequently, the observed differences in average reflectance spectra result from the combined effects of the following factors: higher-grade leaves such as B1F and B2F generally contain higher sugar content and appropriate nicotine levels, leading to greater concentrations of O–H and C–H groups, stronger overall absorption in the 1000–1400 nm region, and thus lower reflectance. In contrast, the relatively lower sugar content in B3F weakens its O–H related absorption, while the higher nicotine in C1F and C2F enhances absorption from C–H and N–H groups. Variations in the concentrations of these specific groups directly determine differences in reflectance intensity at characteristic wavelengths. In summary, the patterns and variations in average reflectance spectra essentially reflect the combined influence of the concentrations of internal chemical groups (O–H, C–H, N–H) and the physical structure of tobacco leaves across different grades.

The lower leaves of first-roasted tobacco had the highest reflectance, followed by the middle leaves, with the upper leaves showing the lowest reflectance. Variations in the structures of tobacco leaves lead to differences in optical behavior. The upper leaves are usually thicker, and the cells are more tightly arranged, which results in reduced light penetration, shorter scattering paths, and lower reflectance. The middle and lower leaves are thinner with a higher proportion of spongy tissues, resulting in increased light scattering and higher reflectance. Significant differences were observed in the chemical compositions of first-roasted tobacco leaves. The Nic content was highest in the upper leaves and lowest in the lower leaves, while the RS and TS contents were lowest in the upper leaves and highest in the middle and lower leaves. Due to the strong absorption characteristics of hydrogen-containing groups (e.g., C–H, O–H, and N–H) in the 1200–1400 nm range, the differences in chemical compositions among leaf positions further amplified variations in their spectral reflectance (Soares et al., 2019). Therefore, different parts and grades of tobacco exhibit distinct spectral responses and characteristic spectral features.

#### 3.3.2 PCA of tobacco leaf sample spectra

**Figure 5:**
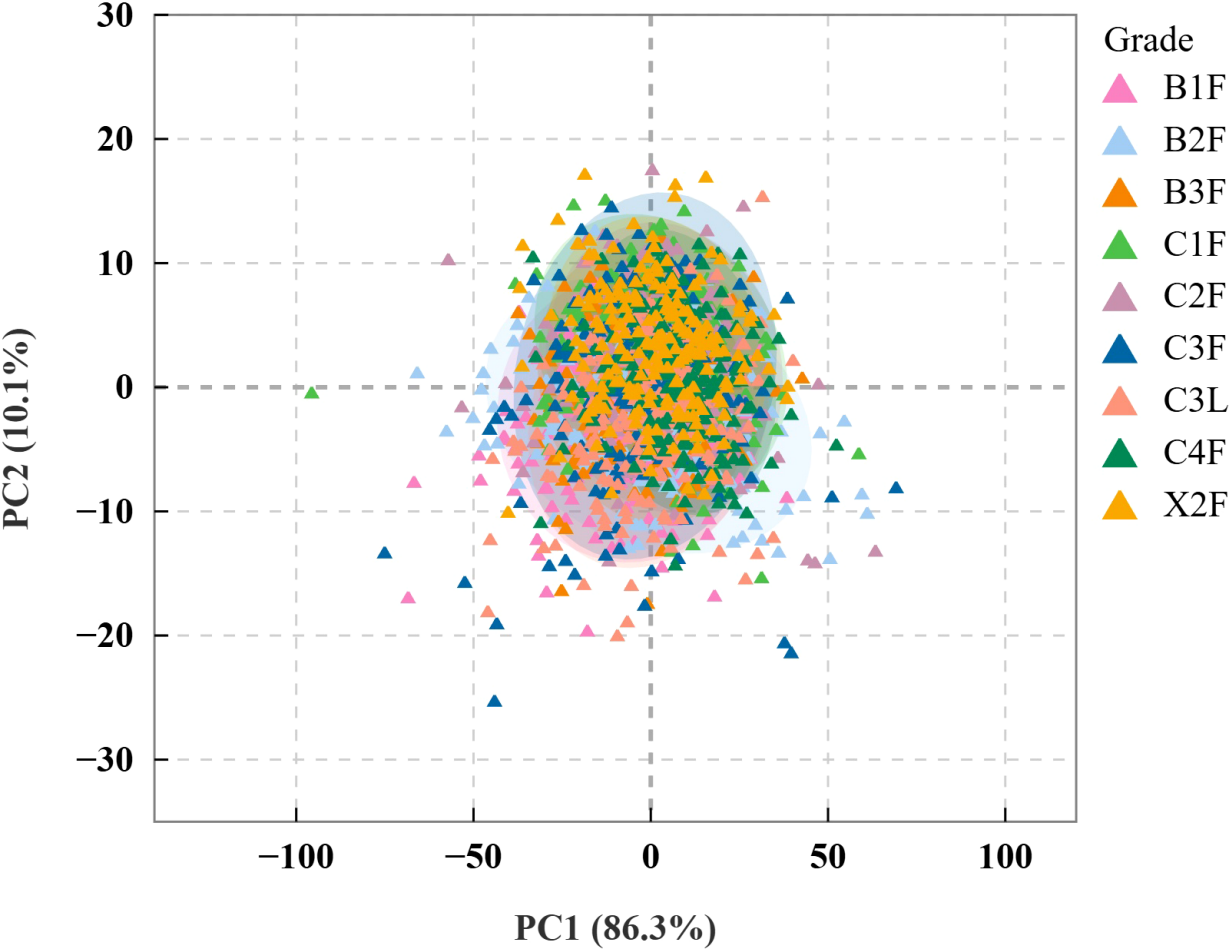
PCA score plot of hyperspectral data for different grades of first-roasted tobacco leaves.

PCA was performed on the collected hyperspectral data of tobacco leaves (**Figure 5**), which showed that the first two principal components cumulatively accounted for 96.4% of the total variance (PC1, 86.3%; PC2, 10.1%). PC1 is the primary factor for distinguishing tobacco leaf grades, encompassing most of the variance and highlighting the pronounced near-infrared hyperspectral differences between leaves of different grades. Near-infrared hyperspectral data have high dimensionality and contain abundant physical and chemical information of flue-cured tobacco leaves, including macroscopic traits such as moisture content, cell structure, and thickness, as well as the chemical information inferred from these characteristics (Marcelo et al., 2019). Additionally, the grades of flue-cured tobacco leaves are classified based on their external physical characteristics, which explains the high contribution rates of the first two principal components. However, PCA could not effectively differentiate between tobacco grades, likely because fluctuations in the O–H bond reflectance peak at 1470 nm, corresponding to water content, were much stronger than the grade-related chemical signals, such as the C–H bond at 1180 nm (Lu et al., 2021b). Moisture content was consistent across all nine grades and did not differ significantly between grades. Therefore, moisture content is unlikely to be a major driver of variation in the PCA or a key factor distinguishing grades. This overshadowed the spectral differences between grades, including those arising from structural variations, preventing PCA from effectively distinguishing tobacco grades.

Although first-roasted tobacco grades showed differences in spectral data and chemical composition, PCA tended to drown out the spectral differences between different grades. The dimensionality of chemical composition data was relatively low, making PCA ineffective for differentiation.

### 3.4 Mantel test analysis of tobacco leaf chemical composition, spectra, and grades

The color of each square in the heat map represents the positive or negative of the correlation coefficient between chemical components. Statistical significance is indicated by asterisks: *** P ≤ 0.001 (extremely significant); ** 0.001 < P ≤ 0.01 (highly significant). The value represents the size of the correlation coefficient. The thickness of the line indicates the strength of the correlation, while the color of the line indicates the degree of salience.

**Figure 6:**
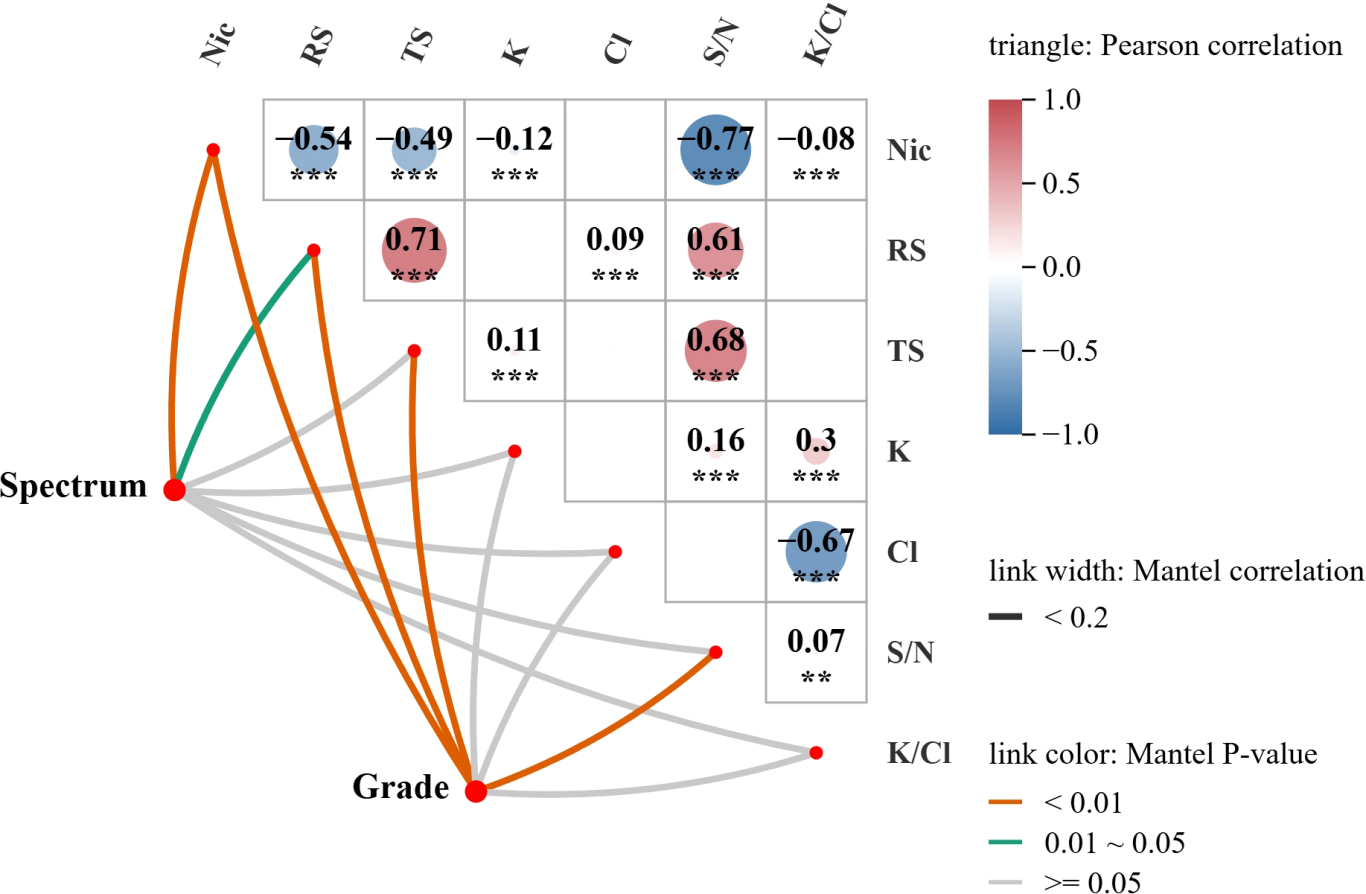
Mantel test analysis of correlations among chemical composition (Nic, RS, TS, K, Cl, S/N, and K/Cl), spectra, and grades of first-roasted tobacco leaves.

Mantel test correlation analysis was performed to explore the relationships between near-infrared hyperspectral data, chemical compositions, and grades of first-roasted tobacco leaves, as well as inter-chemical correlations (**Figure 6**). Core results showed that: (1) Nic exhibited a strong, highly significant negative correlation with RS, TS, and S/N ratio (P ≤ 0.001); (2) Cl^-^ was strongly and highly significantly negatively correlated with K/Cl ratio (P ≤ 0.001); (3) RS and TS showed strong, highly significant positive correlations with each other and with S/N ratio (P ≤ 0.001); (4) Tobacco grade was highly significantly correlated with Nic, RS, TS, and S/N ratio (P < 0.01) but not with K^+^, Cl^-^, or K/Cl ratio (P > 0.05); (5) Hyperspectral data were highly significantly correlated with Nic (P < 0.01), significantly correlated with RS (0.01 < P < 0.05), but not significantly correlated with TS, K^+^, Cl^-^, S/N ratio, or K/Cl ratio (P > 0.05).

The antagonistic relationship between Nic and sugars (RS/TS) is consistent with previous findings that enhanced Nic synthesis consumes sugars or inhibits sugar accumulation in tobacco, leading to a negative correlation between these components (Mo et al., 2022). The strong positive correlation between RS and TS is attributed to RS accounting for 60–80% of TS, a common compositional feature of tobacco carbohydrates (Banožić et al., 2020). As a composite index reflecting carbon-nitrogen metabolic balance, the S/N ratio is jointly regulated by sugars and Nic; its positive correlation with RS/TS and negative correlation with Nic aligns with the well-documented antagonism between carbon and nitrogen metabolism in tobacco (Bush and Tso, 2015).

The non-significant correlation between tobacco grade and K^+^, Cl^-^ (or K/Cl ratio) is explained by the fact that tobacco grading primarily relies on appearance traits (maturity, color, thickness, oil content) rather than mineral content (Li et al., 2024a). K^+^ and Cl^-^ are mainly affected by soil fertility and fertilizer management, with no direct link to appearance traits. In contrast, Nic, RS, and TS are closely associated with appearance quality: sufficient nitrogen supply promotes Nic synthesis, enhancing leaf tissue compactness and color development (Huan et al., 2024); sugars contribute to oiliness and luster via hygroscopic properties and Maillard reactions (generating pigments and aroma compounds during curing) (Wu et al., 2020; Yang et al., 2023); and the S/N ratio correlates strongly with leaf maturity, a key grading criterion (Grsic and Čavlek, 2019).

The spectral correlation patterns are determined by molecular vibrational characteristics. Hyperspectral signals in the near-infrared region (780–1650 nm) originate from C–H, O–H, and N–H functional group vibrations. Nic and RS exhibit detectable characteristic absorption: Nic’s N–H and C–H groups absorb at 1228–1370 nm, while RS’s O–H and C–H groups show distinct near-infrared absorption peaks (Golic et al., 2003; Robertson et al., 2019). In contrast, K^+^ and Cl^-^ are spectrally inert due to the absence of hydrogen-containing functional groups; TS signals are masked by spectral overlap with other hydroxyl-containing compounds (e.g., RS, cellulose, starch) (Qin et al., 2021); and the S/N ratio, as a non-chemical entity, cannot be directly detected due to overlapping signals from sugars and Nic.

The Mantel test correlation analysis reveals the relationships among chemical components, tobacco grades, and spectral data through the following logical chain. The chemical components of tobacco leaves serve as their intrinsic basis, directly determining their physical properties and external appearance. Furthermore, grade serves as an external manifestation of the chemical composition, allowing the intrinsic quality to be visualized through empirical grading standards. Meanwhile, hyperspectral imaging quantifies these grade-related variations in physical traits and chemical composition into hyperspectral data, with the resulting spectral profiles providing a comprehensive reflection of tobacco leaf quality. Using chemical components as the foundational basis, the quantified spectral data and the appearance traits that define grade are integrated, forming an interconnected system that links the spectrum, grade, and chemical components. This provides a foundation for predicting the grades of first-roasted tobacco leaves using hyperspectral technology.

### 3.5 Comparison of spectral preprocessing and model selection

**Figure 7:**
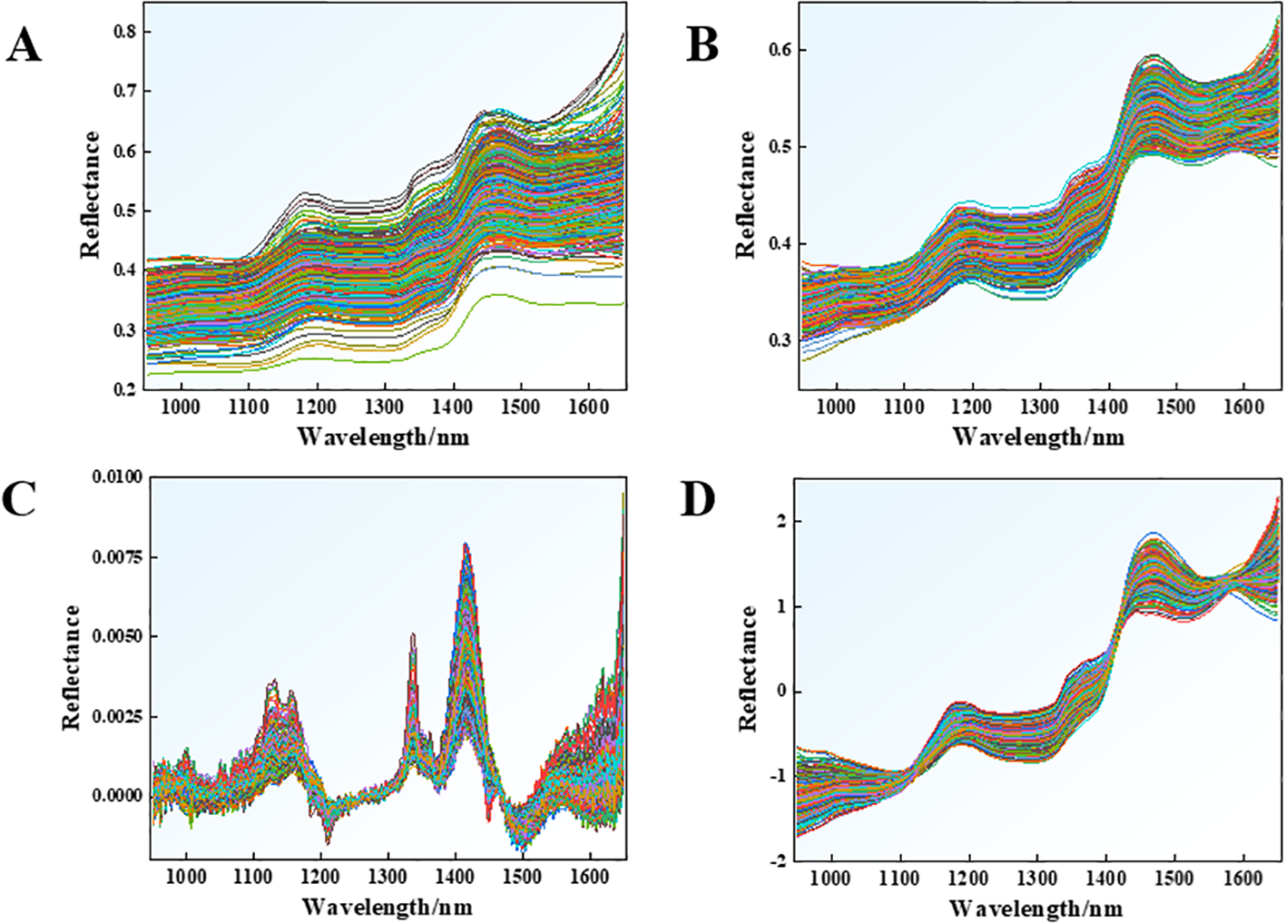
Comparison of raw and preprocessed spectra. **(A)** Original spectra. **(B)** Spectra after MSC preprocessing. **(C)** Spectra after SG preprocessing. **(D)** Spectra after SNV preprocessing.

Three preprocessing methods (namely, MSC, SG, and SNV) were applied to the hyperspectral data of different grades of first-roasted tobacco leaves, and the spectral curves of all samples after preprocessing were plotted (**Figure 7**). After data preprocessing, the spectral curves of first-roasted tobacco leaves were more concentrated than the original spectra. Spectral data processed by both SG and SNV exhibited increased dispersion at the boundary wavelengths, attributed to inherent algorithmic limitations—namely, boundary extrapolation uncertainty for SG and noise amplification in low signal-to-noise regions for SNV (Buddenbaum and Steffens, 2012; Schmid et al., 2022). In contrast, MSC-corrected spectra remained highly consistent with the original spectral profiles.

To establish four classification models (RF, BPNN, ELM, and PLS-DA), the three preprocessed spectral datasets were used as input variables, and the tobacco grades were used as output variables. The correct classification rate of the test set was used as the evaluation index for these models (**Table 2**). SNV-RF had the lowest classification accuracy of only 34.0%, while MSC-PLS-DA had the highest classification accuracy of 98.5%. The lowest average classification accuracy among the three preprocessing methods was 74.6% for SNV, and the highest average classification accuracy was 90.1% for MSC. When first-roasted tobacco grades were categorized using near-infrared hyperspectroscopy, the differences between grades were mainly reflected in the contents of key chemical constituents such as nicotine, total sugars, and reducing sugars (Zhu et al., 2022). MSC enhances chemical compositional differences by removing spectral baseline shifts caused by surface scattering or uneven particle distribution (Li et al., 2024b). SG removes high-frequency noise but has limited capability to correct for systematic noise and does not fully preserve chemical information (Schmid et al., 2022). SNV processes each spectrum individually, which may disrupt the continuity of the chemical gradient between samples (Mishra et al., 2021). As a result, MSC achieves the highest average classification accuracy (Rinnan et al., 2009).

**Table 2:** Accuracy of different preprocessing methods and classification models for tobacco leaf grade identification using full-band spectra.

Model Preprocessing	Grade				Average classification accuracy
	RF	BPNN	ELM	PLS-DA	
MSC	79.8%	93.6%	88.4%	98.5%	90.1%
SG	60.2%	93.5%	95.1%	98.1%	86.7%
SNV	34.0%	89.7%	77.2%	97.4%	74.6%
Average classification accuracy	58.0%	92.3%	86.9%	98.0%	

Comparing the four classification models, the PLS-DA model achieved the best performance with an average classification accuracy of 98.0%, while RF had the lowest average classification accuracy of 58.0%. Hyperspectral data are characterized by high dimensionality and strong collinearity. PLS-DA, which combines PCA and partial least squares regression, can effectively extract features relevant to the classification target while reducing data dimensionality, thereby improving classification accuracy. During hyperspectral scanning, instrumental noise, baseline drift, and other issues can occur. RF is sensitive to noise, while BPNN and ELM tend to amplify noise in the low signal-to-noise ratio regions, resulting in decreased classification stability for all three models (Agjee et al., 2018; Huang et al., 2019). Considering model accuracy and differences in classification performance, MSC was chosen as the preprocessing method, and PLS-DA was selected as the classification model.

### 3.6 Comparison of characteristic band algorithms

**Figure 8:**
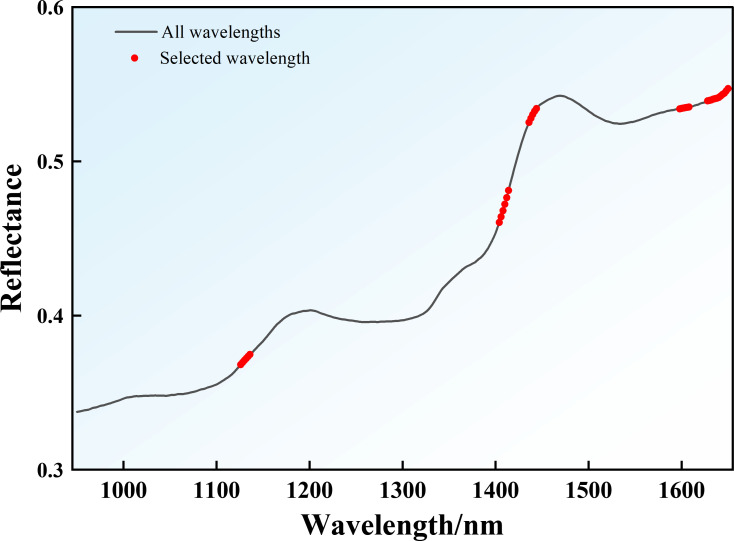
CARS characteristic band distribution.

When an excessive number of bands is selected (e.g., more than 50 bands, corresponding to >15% of the original set), the retained redundant information tends to obscure the inherent differences in the feature selection preferences of the two algorithms. Conversely, an insufficient number of bands (e.g., fewer than 20 bands, or <6% of the original set) leads to the loss of critical spectral information, resulting in a “floor effect” that would diminish their informativeness (Tang et al., 2014; Li et al., 2024c). Therefore, 35 feature wavelengths, accounting for 10% of the original spectral data, were selected to compare the performance of the SPA and CARS algorithms. The CARS algorithm selected characteristic wavelengths primarily concentrated around 1130 nm, 1410 nm, 1440 nm, 1600 nm, and 1630–1650 nm, which are distributed across the entire near−infrared spectral range (**Figure 8**). These bands correspond to multiple chemical−bond vibration signals associated with key tobacco components: the band near 1130 nm (attributed to overtones and combination tones of C–H bonds) primarily reflects carbohydrate content (Zhou et al., 2025); the bands at 1410 nm and 1440 nm (resulting from combination vibrations of O–H and C–H bonds) specifically indicate the content and structural features of soluble sugars in tobacco (Li et al., 2024a); the bands around 1600 nm and 1630–1650 nm (associated with C–H and N–H bond vibrations) reflect variations in Nic content (Liang et al., 2022). Thus, CARS effectively integrates spectral information related to sugars and Nic.

**Figure 9:**
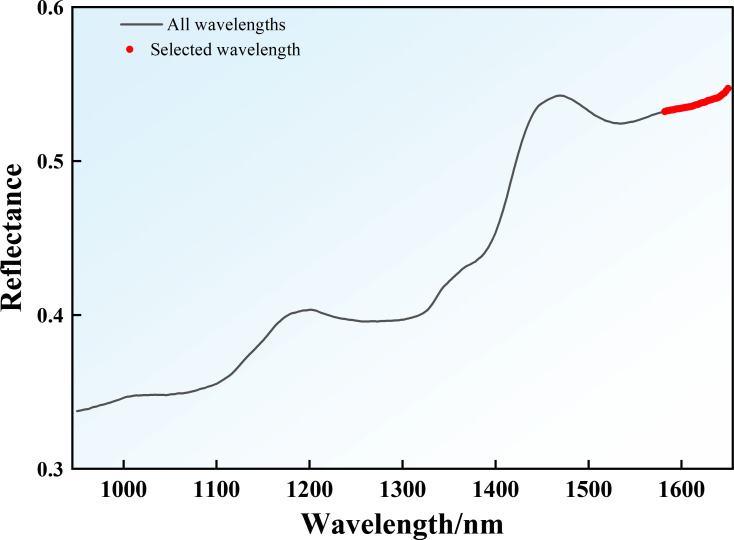
SPA characteristic band distribution.

In contrast, SPA predominantly selected wavelengths within the 1580–1650 nm range (**Figure 9**), which corresponds to combination vibrations of C–H bonds. This spectral region is less affected by light scattering caused by cellular structures, thereby minimizing physical interference and enhancing the absorption signals of C–H-containing constituents such as sugars and Nic (Zhu et al., 2022). Consequently, these wavelengths serve as highly informative characteristic bands for characterizing the chemical profile of tobacco.

In summary, both algorithms successfully reduced the dimensionality of the hyperspectral data while retaining key spectral information linked to the core chemical constituents of tobacco, thereby achieving a balance between model fitting performance and generalization capability.

### 3.7 Model evaluation and analysis

#### 3.7.1 PLS-DA classification model

The PLS-DA model demonstrates consistently higher grade classification accuracy than other classification models across all three preprocessing methods, achieving an accuracy of 98.5% under MSC preprocessing. This is because there is strong collinearity among the bands in the near-infrared spectra. PLS-DA, however, extracts latent variables by maximizing covariance, automatically filtering out noise and emphasizing discriminative bands. Thus, the classification accuracy of PLS-DA is significantly higher than that of the other three models.

**Figure 10:**
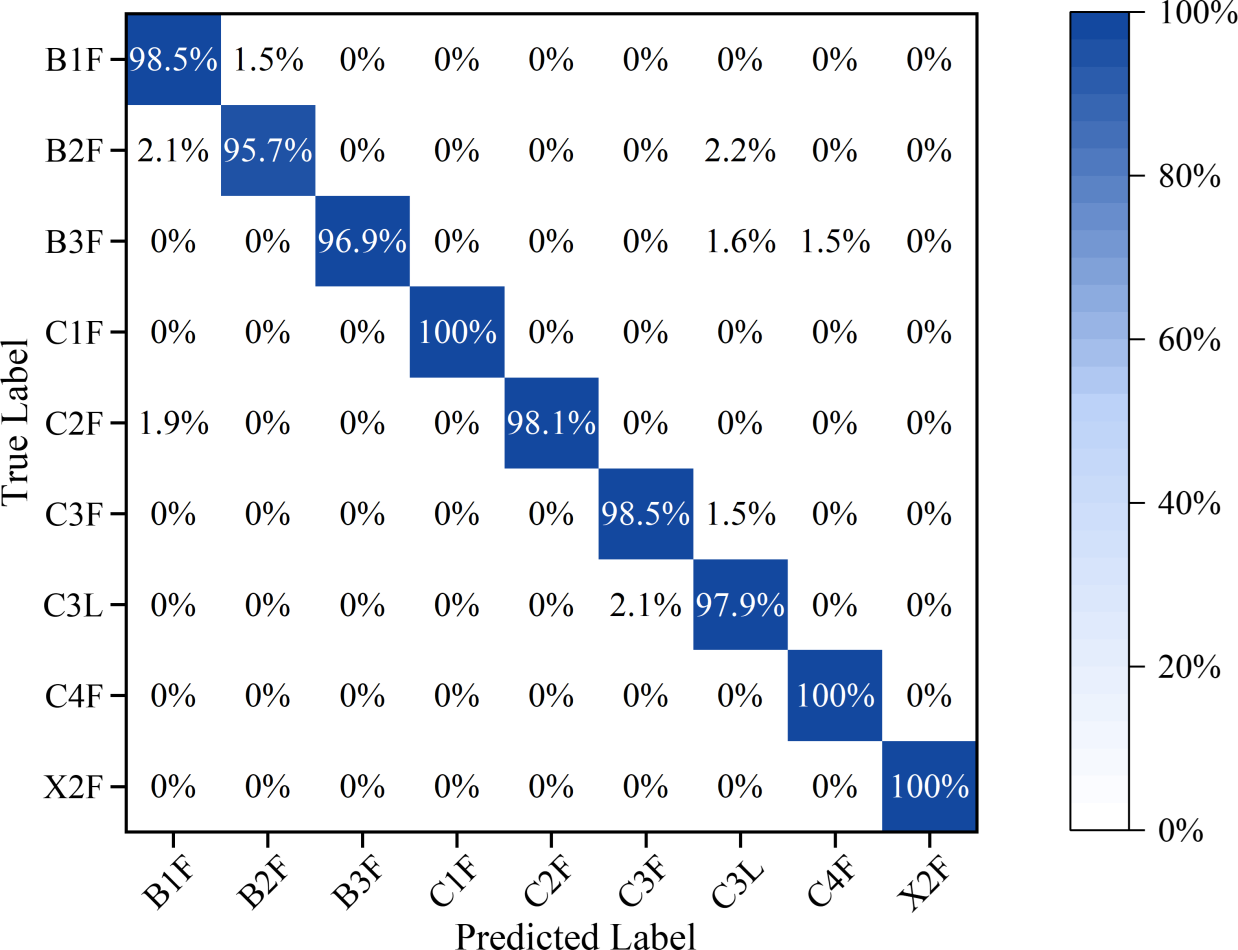
Confusion matrix for the hierarchical classification of PLS-DA models.

The experiment employed the confusion matrix as the performance evaluation index for the model (**Figure 10**). Values along the diagonal of the confusion matrix represent the proportion of correctly categorized samples, while values on the off-diagonal denote the proportion of incorrectly categorized samples. The classification accuracy for C1F, C4F, and X2F reached 100%, while that for B1F, C2F, and C3F averaged 98.0%, with only a very small portion of samples misclassified. B2F is similar to B1F in both appearance and intrinsic chemical composition, and the near-infrared spectra primarily reflect the overall chemical information of tobacco leaves. If different grades of tobacco differ only slightly in key components, their spectral features may overlap in the principal component space, making it difficult for the model to distinguish them. As a result, 2.1% of B2F samples were miscategorized as B1F. The slight differences in the physical structures of C3L and C3F in the middle leaves were not sufficiently reflected in the near-infrared spectra, leading to 2.1% of C3L samples being classified as C3F. Although a small number of misclassifications occurred, the confusion matrix demonstrates that the MSC-PLS-DA model has strong classification performance, accurately distinguishing between tobacco grades.

#### 3.7.2 Comparison of different numbers of characteristic bands

The MSC-preprocessed data were input into the SPA and CARS characteristic band selection algorithms, which selected varying numbers of characteristic bands. These numbers were then placed in ascending order according to the ratio of characteristic bands to the full wavelength range and fed into the grade classification model to evaluate the classification accuracy for different band counts. In the SPA, the classification accuracy of the RF model does not increase significantly as the number of feature bands increases (**Table 3**). The remaining three models have unsatisfactory performance when the number of feature bands is small. However, the classification accuracy of all three models improves as the number of feature bands increases, increasing by 20% for ELM and by 40% for BPNN and PLS-DA. In the CARS algorithm, the classification performance of all four models is unsatisfactory when the number of feature bands is small (**Table 4**). Similar to the SPA, the classification accuracy of all four models improves as the number of feature bands in the CARS model increases, increasing by 40% for RF, by 30% for BPNN and PLS-DA, and by 10% for ELM. When the number of selected feature bands is limited, the wavelengths chosen by SPA are predominantly concentrated in the spectral end regions. RF owing to its ensemble learning mechanism, exhibits strong robustness to locally concentrated high-information features, enabling effective extraction of discriminative information from such intervals (Bin et al., 2016). In contrast, both BPNN and ELM rely on nonlinear correlations among multiple constituent features, while PLS-DA depends on linear relationships across multiple bands to construct meaningful latent variables (Jiang et al., 2017; Shao et al., 2022). A single spectral interval is insufficient to meet the input requirements of these models, leading to relatively lower classification accuracy. Although the wavelengths selected by CARS cover key spectral regions associated with sugars and nicotine, its selection bias—rooted in the regression coefficients of PLS and compounded by the randomness introduced through Monte Carlo sampling—tends to discard signal bands that are critical for classification (Song et al., 2020). Consequently, the resulting feature subset may lack representativeness, hindering the ability of all classification models to extract effective discriminatory information. Therefore, when the number of feature bands is limited, all classification models based on bands selected by SPA (with the exception of the RF model) or CARS fail to achieve satisfactory classification accuracy.

**Table 3:** Number of SPA-selected feature bands and the corresponding hierarchical classification accuracy across different models.

Number of characteristic bands (pieces)	Accuracy (%)			
	RF	BPNN	ELM	PLS-DA
35 (10%)	76.1	58.9	69.2	46.0
70 (20%)	77.0	69.0	73.3	73.8
105 (30%)	76.3	71.0	78.7	81.9
140 (40%)	75.5	76.8	81.1	88.8
175 (50%)	76.8	79.4	83.0	91.6
210 (60%)	77.8	81.7	82.1	93.6
245 (70%)	77.9	84.5	86.7	94.0
280 (80%)	77.6	86.7	85.1	95.5
315 (90%)	79.6	92.5	87.1	96.3

**Table 4:** Number of CARS-selected feature bands and the corresponding hierarchical classification accuracy across different models.

Number of characteristic bands (pieces)	Accuracy (%)			
	RF	BPNN	ELM	PLS-DA
35 (10%)	35.1	50.3	74.6	61.5
70 (20%)	59.3	64.1	75.3	77.0
105 (30%)	62.1	72.7	78.9	83.7
140 (40%)	63.6	78.5	84.9	92.0
175 (50%)	67.7	75.7	86.4	91.6
210 (60%)	70.7	82.8	87.5	91.8
245 (70%)	72.5	83.6	86.5	94.4
280 (80%)	77.4	82.1	85.4	93.1
315 (90%)	79.6	87.1	88.0	96.8

In both BPNN and PLS-DA models, the use of characteristic band algorithms results in a significant improvement in classification accuracy as the number of characteristic bands increases. The performance of the BPNN and PLS-DA models is influenced by the information density and signal-to-noise ratio of the input features, while the SPA and CARS algorithms optimize feature subsets, significantly improving data quality and thereby enhancing model performance. Specifically, the SPA, with its low collinearity and small-scale feature subsets, aligns with the sensitivity of BPNN and the projection requirements of PLS-DA, thus significantly improving classification accuracy. In contrast, the CARS algorithm retains a broad range of wavelengths, making it more suitable for tasks requiring comprehensive spectral information. After applying the SPA characteristic wavelength selection method, the accuracy of the SPA-RF model remains essentially unchanged. When the number of selected feature bands reached 70% of the total spectral bands (i.e., 246 bands), the classification accuracy of the SPA-ELM and SPA-PLS-DA models decreased by only 1.7% and 4.5%, respectively, compared to the full-band models. These results demonstrate that a 30% reduction in data dimensionality (i.e., 105 bands)—achieved at the cost of a marginal drop in accuracy—significantly lowers the computational load and storage requirements of the models. This feature selection strategy effectively avoids the redundancy associated with full-band modeling while addressing the performance limitations of low-dimensional feature sets. It thus offers a practical and feasible solution for the lightweight deployment and engineering application of hyperspectral-based tobacco grading technology.

The PLS-DA model achieves a significantly higher classification accuracy than the other three models when classifying grades based on both full bands and selected feature bands. When the number of feature bands reaches 70% of the total, the classification accuracy of the PLS-DA model is comparable to that achieved using the full set of bands, indicating that PLS-DA is well suited for tobacco grade classification. In this study, a model developed by integrating hyperspectral technology with machine learning has classified nine different grades of first-roasted tobacco with an accuracy of 98.5%, achieving accurate discrimination across different parts and grades.

Compared with previous studies, this research mainly focuses on analyzing the near-infrared hyperspectral data of nine common grades of flue-cured tobacco leaves. Wei et al., 2024 used the 1D-CNN model combined with the LAR characteristic band algorithm to classify 10 grades of flue-cured tobacco leaves, achieving an accuracy of 96.3%. Similarly, Lu et al., 2021a used CNN to classify tobacco leaf grades based on global images and local patches, with a classification accuracy of 91.3%. In contrast, the classification accuracy of this study reached 98.5%, representing improvements of 2.2% and 7.2% over the aforementioned studies of Wei et al., 2024 and Lu et al., 2021a respectively. Furthermore, Wei et al., 2024 used the visible–near-infrared (401–1046 nm) spectral range, while Lu et al., 2021a performed a classification based on tobacco leaf images. This study has employed near-infrared hyperspectroscopy (950–1650 nm), which provides greater stability and richer spectral information, making it more suitable for classifying tobacco leaf grades.

In data-driven models for agricultural product quality classification, variations in ecological conditions, cultivation practices, and inter-annual environmental fluctuations across different regions can lead to shifts in spectral features. The core challenge arising from this phenomenon is that when a model is directly applied to crops from other regions or subsequent years, it may encounter novel feature patterns not sufficiently represented in the training data, consequently leading to a degradation in classification performance (Wang et al., 2025). Although the classification model developed in this study demonstrates excellent performance on the experimental samples, it is crucial to acknowledge this inherent limitation. The generality and robustness of the model are not absolute; its long-term and broad applicability relies on a sustained update mechanism. This entails continuously incorporating new samples from diverse regions and harvest years to recalibrate and retrain the model, enabling it to dynamically adapt to the spatiotemporal variability in tobacco leaf spectral characteristics. This limitation is not unique to our model but represents a common challenge that all data-driven agricultural product quality classification models must address to transition successfully into practical application (Wang et al., 2025).

## 4 Conclusion

In this study, hyperspectral imaging technology was combined with machine learning methods to classify the grades of first-roasted tobacco, and the intrinsic relationships among grade, spectrum, and chemical composition were explored through multivariate statistical analysis. Mantel test correlation analysis revealed significant correlations among spectral data, tobacco grades, and chemical components. Chemical components constitute the intrinsic basis that determines the external characteristics and grades of tobacco leaves. By capturing the vibrational information of hydrogen-containing groups, spectra quantify the physical and chemical characteristics associated with tobacco grades, serving as a bridge between grades and chemical components and providing theoretical support for hyperspectral-based grading. The MSC, SG, and SNV preprocessing methods were combined with the RF, BPNN, ELM, and PLS-DA classification models to classify first-roasted tobacco leaves by grade. Additionally, the SPA and CARS feature band algorithms were used to extract feature bands from the spectral data. The classification accuracy of MSC-PLS-DA reached 98.5%. Employing only 70% of the feature bands (245 bands), the SPA-ELM and SPA-PLS-DA models maintained classification accuracies of 86.7% and 94.0%, on par with the full-band models (88.4% and 98.5%, respectively), while markedly reducing computational overhead and storage needs. Notably, the selected bands concentrated on the key spectral intervals corresponding to tobacco chemical components, effectively eliminating redundant information and achieving a sound balance between classification accuracy and model efficiency. This study has not only elucidated the multivariate relationships among spectra, chemical components, and grades but also provided an efficient method for the grading of flue-cured tobacco leaves. Additionally, it has provided a solid theoretical basis and mechanistic explanation for the application of hyperspectral technology in practical industrial settings and established a foundation for efficient tobacco leaf grading across the industry, facilitating the realization of automated grading and enhancing the economic benefits of the tobacco sector.

## Data Availability

The raw data supporting the conclusions of this article will be made available by the authors, without undue reservation.
