# Supplementary material for: Enabling rapid and accurate grand discrimination of flue-cured tobacco: a near-infrared hyperspectral and machine learning approach
Source: Front Plant Sci. 2026 Feb 24;17:1756218. doi: 10.3389/fpls.2026.1756218 (PMC12974139; doi:10.3389/fpls.2026.1756218)
Supplement: Supplementary file 1 [file Table1.docx]

**SUPPLEMENTARY INFORMATION**

**Enabling rapid and accurate grand discrimination of flue-cured tobacco: a near-infrared hyperspectral and machine learning approach**

Jiang Zou^1^^,2†^, Hongbo Gao^2†^, Duo Wang^3^, Yunquan Chen^2^, Shiyou Deng^3^, Nuo Shi^2^, Shengjie Yang^2^, Chunlin Huang^2^, Dingchun Zi^2^, Yu Du^2^, Yuxiang Bai^2^, Na Wang^2^, Ge Wang^2^, Zhengling Liu^3*^, Junhua Zhang^1*^, Peng Zhou^2*^

^1^ Kunming University of Science and Technology, Kunming, Yunnan, 650500, China

^2^ Yunnan Agricultural University, Kunming, Yunnan, 650201, China

^3^ Kunming Branch of Yunnan Tobacco Company, Kunming, Yunnan, 650051, China

**Total pages: 3**

**Table: 1**

**List of supporting information**

Table

Table S1. Information on samples of different grades of tobacco leaves

**Tab. S1.** Information on samples of different grades of tobacco leaves

| **Tobacco grade** | **Number of samples** | **Parts** | **Quality level** | **Appearance description** |
| --- | --- | --- | --- | --- |
| orange leaf grade one (B1F) | 206 | leaf | highest quality | Maturity ripe; leaf structure firm; body fleshy; oil rich; color intensity deep |
| orange leaf grade two (B2F) | 197 | leaf | highest quality | Maturity rip; leaf structure firm; body fleshy; oil oily; color intensity strong |
| orange leaf grade three (B3F) | 204 | leaf | medium quality | Maturity rip; leaf structure close; body fleshy; oil oily; color intensity moderate |
| orange cutters grade one (C1F) | 195 | cutters | highest quality | Maturity rip; leaf structure open; body medium; oil rich; color intensity deep |
| orange cutters grade two (C2F) | 200 | cutters | highest quality | Maturity rip; leaf structure open; body medium; oil oily; color intensity strong |
| orange cutters grade three (C3F) | 196 | cutters | highest quality | Maturity rip; leaf structure open; body medium; oil oily; color intensity moderate |
| lemon cutters grade three (C3L) | 198 | cutters | medium quality | Maturity rip; leaf structure open; body less thin; oil oily; color intensity moderate |
| orange cutters grade four (C4F) | 188 | cutters | medium quality | Maturity rip; leaf structure open; body less thin; oil less oily; color intensity moderate |
| orange lugs grade two (X2F) | 200 | lugs | medium quality | Maturity rip; leaf structure open; body less thin; oil less oily; color intensity moderate |
